# Supplementary material for: Identification of the Anti-Infective Aborycin Biosynthetic Gene Cluster from Deep-Sea-Derived Streptomyces sp. SCSIO ZS0098 Enables Production in a Heterologous Host
Source: Mar Drugs. 2019 Feb 21;17(2):127. doi: 10.3390/md17020127 (PMC6409603; doi:10.3390/md17020127)
Supplement: Supplementary file 1 [file marinedrugs-17-00127-s001.pdf]

Supporting Information

Identification of the Anti-infective Aborycin Biosynthetic Gene Cluster from Deep-Sea-Derived *Streptomyces* sp. SCSIO ZS0098 Enables Production in a Heterologous Host

Mingwei Shao[1,2] Juying Ma,[1,2] Qinglian Li,[1,2] and Jianhua Ju[1,2,*]

[1] CAS Key Laboratory of Tropical Marine Bio-resources and Ecology, Guangdong Key Laboratory of Marine Materia Medica, RNAM Center for Marine Microbiology, South China Sea Institute of Oceanology, Chinese Academy of Sciences, 164 West Xingang Road, Guangzhou 510301, China; jianting880720@126.com (M.S.); majunying@scsio.ac.cn (J. M.); liql@scsio.ac.cn (Q. L.)

[2] University of Chinese Academy of Sciences, 19 Yuquan Road, Beijing 110039, China

* Correspondence: jju@scsio.ac.cn (J.J.); Tel./Fax: +86-20-3406-6449 (H.H.); Tel./Fax: +86-20-8902-3028 (J.J.)

Supporting Information Table of Contents

Description of Supporting Item	Page
**Table S1.** Bacteria used in this study	**S3**
**Table S2**. Plasmids used in this study	**S3**
**Table S3.** Primers used in this study	**S3**
**Figure S1.** Positive ion peak for mass spectra of analytically pure aborycin.	**S4**
**Figure S2.** Tandem mass analysis and derived structure of a peptide (parent ion $[M + H]^+$ at m/z = 887.3451) yielded from acid hydrolysate of aborycin.	**S5**
**Figure S3**. LC-MS analysis of aborycin accumulated in the cultures of *Streptomyces coelicolor* M1152/1512H.	**S6**
**Figure S4** ^{1}H NMR (500 MHz) spectrum of aborycin in DMSO-d_6.	**S7**
**Figure S5** ^{13}C NMR (125 MHz) spectrum of aborycin in DMSO-d_6.	**S8**
**References**	**S9**

Table S1. Bacteria used in this study.

Strains	Description	Source/[Ref]
E. coli		
DH5α	Host strain for general clone	
BW25113/pIJ790	K-12 derivative: *araBAD*, *rhaBAD*; host strain for Red/ET-mediated recombination	[1]
ET12567/pUZ8002	*dam*, *dcm*, *hsdM*, *hsdS*, *hsdR*, *catR*, *tetR*; donor strain for conjugation between *E. coli* and *Streptomyces*	[2]
XL 1-Blue MR	Host strain for construction of genomic cosmid library	Stratagene
**Streptomycetes**		
Streptomyces coelicolor M1152	Host strain for heterologous expression of the *abm* biosynthetic gene cluster	
Streptomyces coelicolor TK64	Host strain for heterologous expression of the *abm* biosynthetic gene cluster	
Streptomyces sp. SCSIO ZS0098	Wild-type producer of aborycin	This work

Table S2. Plasmids used in this study.

Plasmids	Description	Source/[Ref]
pIJ773	P1-FRT-oriT-aac(3)IV-FRT-P2	[3]
pIJ790	λ–RED (*gam bet exo*) CmlR *araCrep101ts*	[4]
pSET152AB	*aac(3)IV-oriT-intϕ*C31	
SuperCos1	Used for construction of genomic cosmid library	Stratagene
cosmid 15-12H	A cosmid which contains partial *abo* biosynthetic cluster	This work

Table S3. Primers used in this study.

Primer Name	Sequence (5'→3')	purpose
screen-orf(-3)-F	TGGAAGCCCGTCACCGCTA	For the screening of the genomic library
screen-orf(-3)-R	CGAACGCACCCACCAGAA	
screen-orf(+3)-F	TGGAAGCCCGTCACCGCTA	For the screening of the genomic library
screen-orf(+3)-R	CGAACGCACCCACCAGAA	
screen-aboB1-F	CGACTTCGGCTGGGTGTTCG	For the screening of the genomic library
screen-aboB1-R	TCGGCTGGAGGTGATGGGAC	

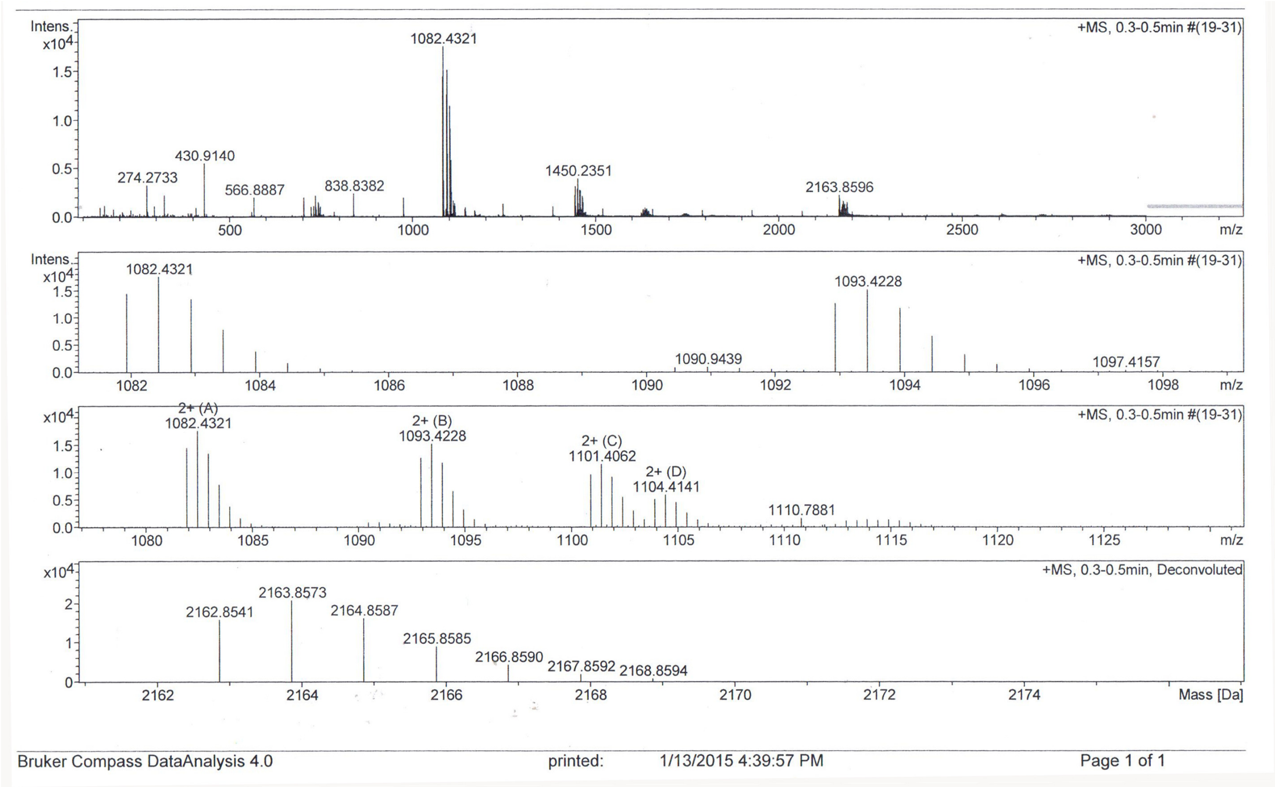

Figure S1. Positive ion peak of mass spectra of aborycin.

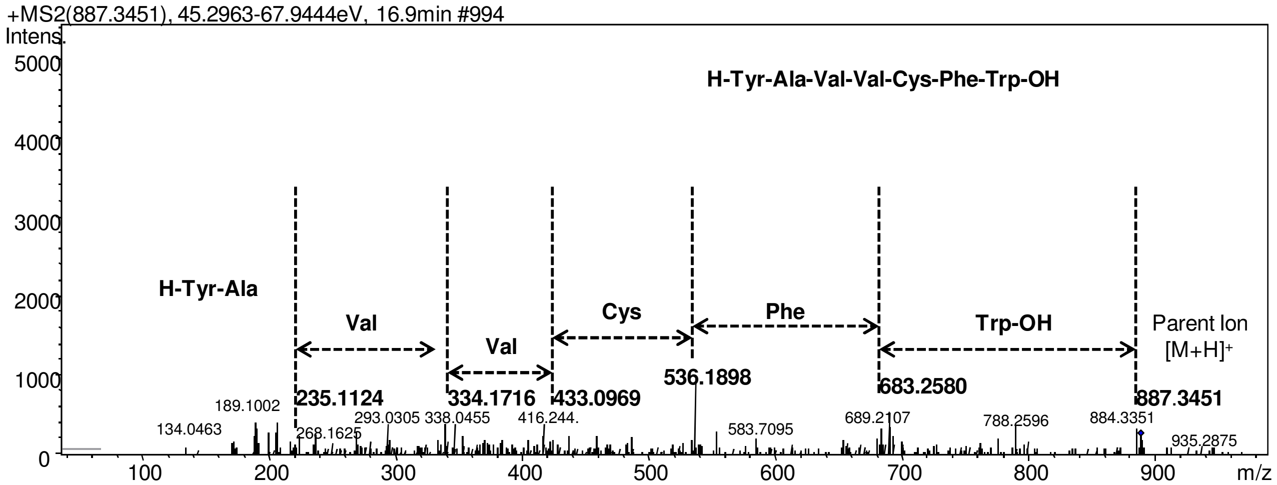

Figure S2. Tandem mass analysis and derived structure of a peptide (parent ion $[M + H]^+$ at m/z = 887.3451) yielded from acid hydrolysate of aborycin.

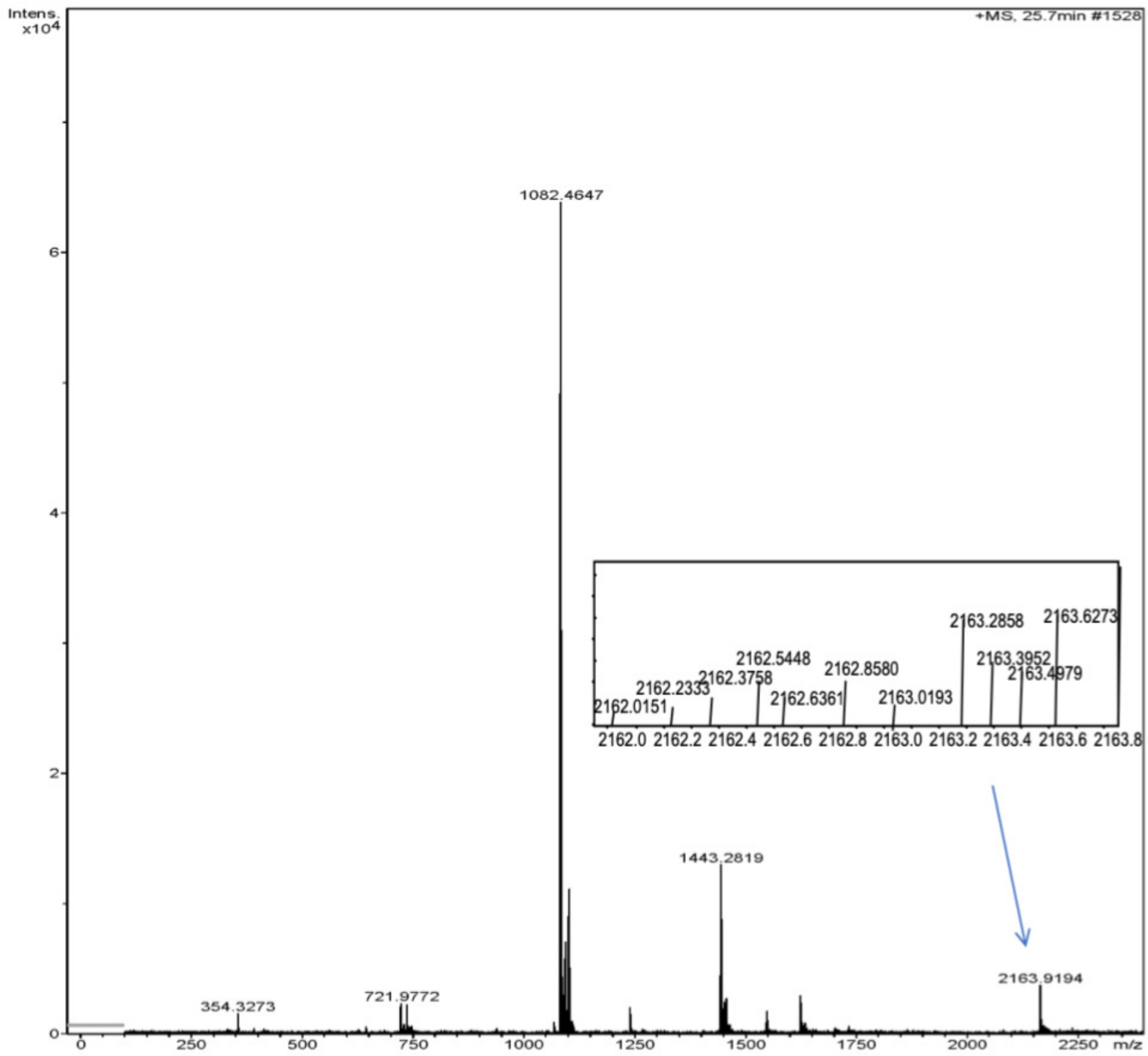

Figure S3. LC-MS analysis of aborycin accumulated in the cultures of *Steptomyces coelicolor* M1152/1512H (Positive mode).

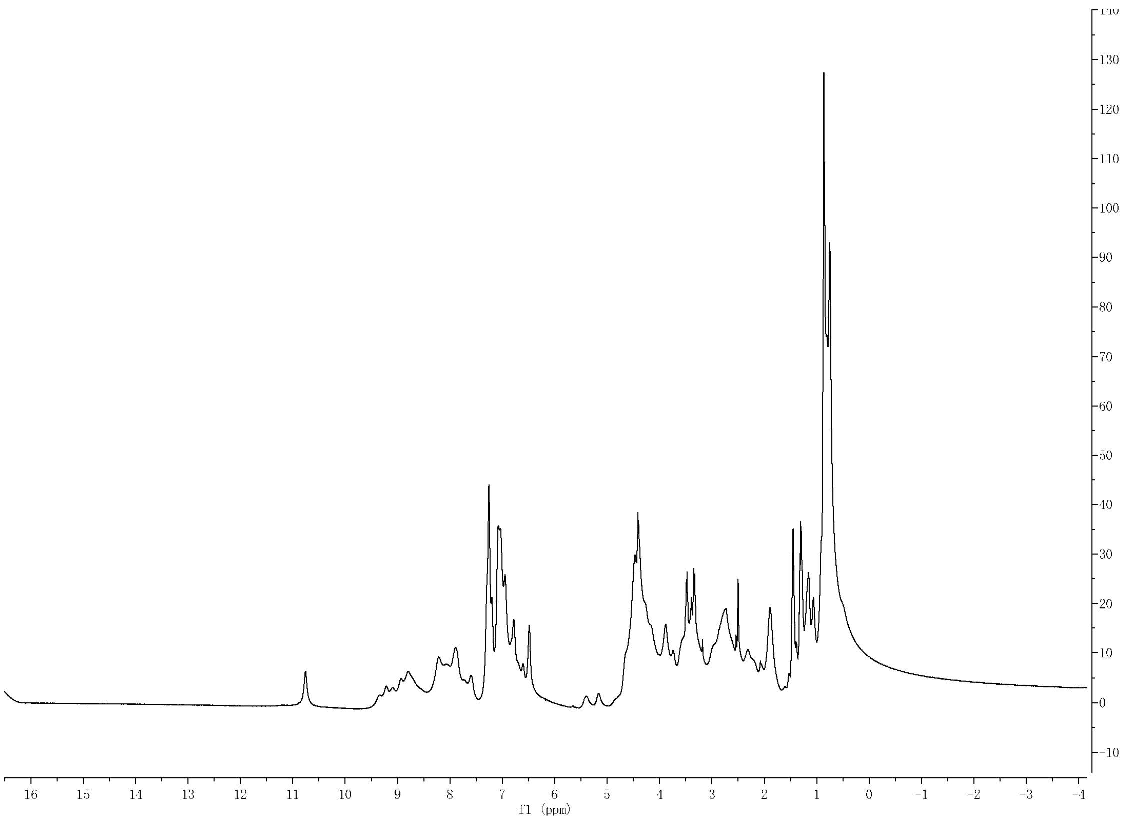

Figure S4. ^{1}H NMR (500 MHz) spectrum of aborycin in DMSO-d_6.

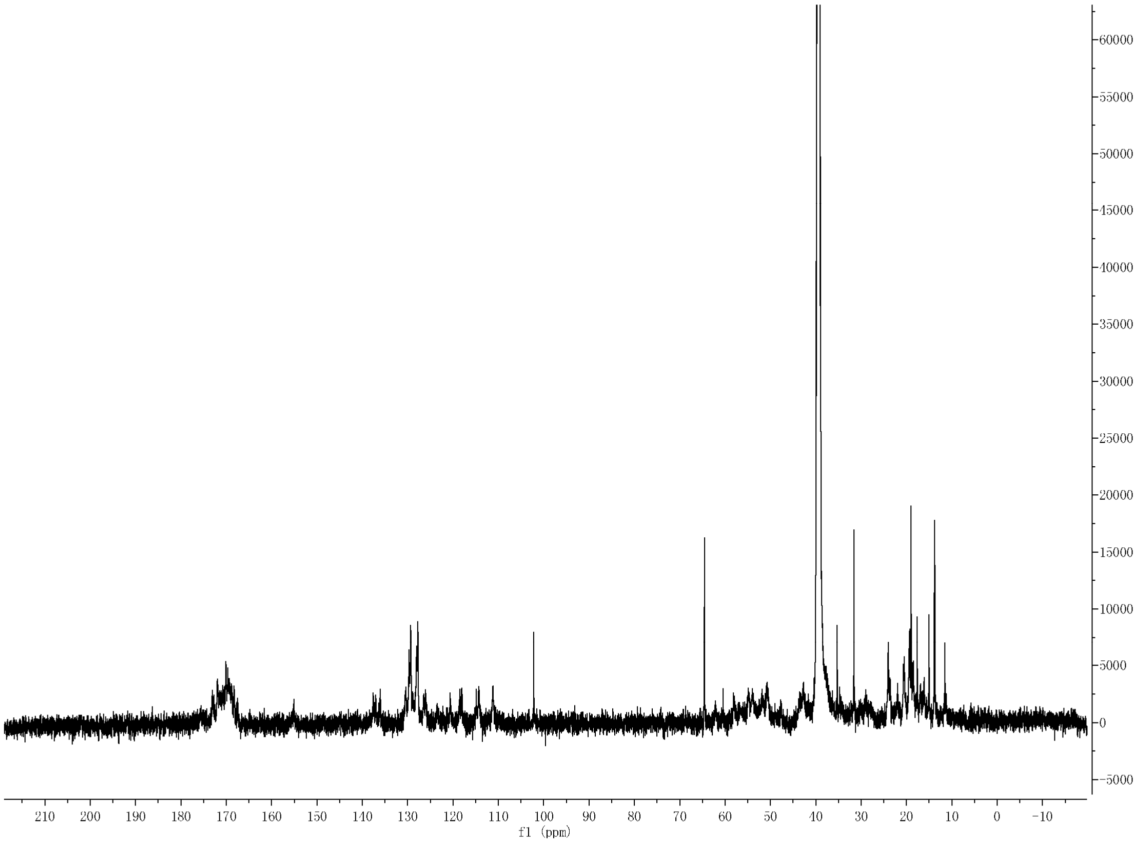

Figure S5. ^{13}C NMR (125 MHz) spectrum of aborycin in DMSO-d_6.

References

1. Datsenko KA, Wanner BL. One-step inactivation of chromosomal genes in Escherichia coli K-12 using PCR products. *Proc. Natl. Acad. Sci. U S A*. **2000**, *97*, 6640–5.

2. MacNeil DJ, Gewain KM, Ruby CL, Dezeny G, Gibbons PH, MacNeil T. Analysis of *Streptomyces avermitilis* genes required for avermectin biosynthesis utilizing a novel integration vector. *Gene*. **1992**, *111*, 61–8.

3. Gust B, Challis GL, Fowler K, Kieser T, Chater KF. PCR-targeted Streptomyces gene replacement identifies a protein domain needed for biosynthesis of the sesquiterpene soil odor geosmin. *Proc Natl Acad Sci U S A*. **2003**, *100*, 1541–6.

4. Hong B, Phornphisutthimas S, Tilley E, Baumberg S, McDowall KJ. Streptomycin production by *Streptomyces griseus* can be modulated by a mechanism not associated with change in the adpA component of the A-factor cascade. *Biotechnol. Lett*. **2007**, *29*, 57–64.
